# Effects of propofol and sevoflurane on hepatic blood flow: a randomized controlled trial

**DOI:** 10.1186/s12871-020-01150-3

**Published:** 2020-09-22

**Authors:** Jurgen van Limmen, Piet Wyffels, Frederik Berrevoet, Aude Vanlander, Laurent Coeman, Patrick Wouters, Stefan De Hert, Luc De Baerdemaeker

**Affiliations:** 1 Department of Anaesthesiology and Perioperative Medicine, Ghent University Hospital, Corneel Heymanslaan 10, 9000 Ghent, Belgium; 2 Department of General and Hepatic-pancreatico-biliary Surgery and Liver transplantation, Ghent University Hospital, Corneel Heymanslaan 10, Ghent, 9000 Belgium

**Keywords:** Propofol, Sevoflurane, Liver circulation

## Abstract

**Background:**

Maintaining adequate perioperative hepatic blood flow (HBF) supply is essential for preservation of postoperative normal liver function. Propofol and sevoflurane affect arterial and portal HBF. Previous studies have suggested that propofol increases total HBF, primarily by increasing portal HBF, while sevoflurane has only minimal effect on total HBF. Primary objective was to compare the effect of propofol (group P) and sevoflurane (group S) on arterial, portal and total HBF and on the caval and portal vein pressure during major abdominal surgery. The study was performed in patients undergoing pancreaticoduodenectomy because - in contrast to hepatic surgical procedures - this is a standardized surgical procedure without potential anticipated severe hemodynamic disturbances, and it allows direct access to the hepatic blood vessels.

**Methods:**

Patients were randomized according to the type of anesthetic drug used. For both groups, Bispectral Index (BIS) monitoring was used to monitor depth of anesthesia. All patients received goal-directed hemodynamic therapy (GDHT) guided by the transpulmonary thermodilution technique. Hemodynamic data were measured, recorded and guided by Pulsioflex™. Arterial, portal and total HBF were measured directly, using ultrasound transit time flow measurements (TTFM) and were related to hemodynamic variables.

**Results:**

Eighteen patients were included. There was no significant difference between groups in arterial, portal and total HBF. As a result of the GDHT, pre-set hemodynamic targets were obtained in both groups, but MAP was significantly lower in group S (*p* = 0.01). In order to obtain these pre-set hemodynamic targets, group S necessitated a significantly higher need for vasopressor support (*p* < 0.01).

**Conclusion:**

Hepatic blood flow was similar under a propofol-based and a sevoflurane-based anesthetic regimen. Related to the application of GDHT, pre-set hemodynamic goals were maintained in both groups, but sevoflurane-anaesthetized patients had a significantly higher need for vasopressor support.

**Trial registration:**

Study protocol number is AGO/2017/002 – EC/2017/0164. EudraCT number is 2017–000071-90.Clin.trail.gov,NCT03772106, Registered 4/12/2018, retrospective registered.

## Background

Maintaining adequate perioperative hepatic blood flow supply is essential for preservation of postoperative normal liver function, especially during major hepatic surgery [1] and liver transplantation for both graft [2–4] and patient [5, 6] survival. HBF is unique because it receives a dual blood flow from both the hepatic artery and the portal vein [7–9]. Regulation of the HBF is complex and depends on many factors [9–11]. As a consequence, any pharmacological intervention may critically interfere with this complex control [12]. Surprisingly, the clinical impact of any pharmacological modulation of the hepatic circulation remains ill-defined. This includes the potential effects of routinely used anesthetic agents, such as propofol and sevoflurane.

Anesthetic agents have been shown to influence HBF [7]. Results from animal studies suggested that both volatile and intravenous anesthetic agents modulate HBF. Animal studies have indicated that propofol increases total HBF. This increase seemed primarily related to the increased portal HBF [13–15]. Only one human study has observed similar effects of propofol on hepatic circulation [16].

The effects of sevoflurane on HBF remain unclear. All volatile anesthetics reduce mean arterial blood pressure (MAP) and cardiac output (CO) in a dose-dependent manner. This has an effect on hepatic circulation. Studies in dogs showed no effect of sevoflurane on total HBF but it was assumed that sevoflurane reduced portal HBF, resulting in a reactive increase of arterial HBF [13, 17, 18].

Based on these data, we hypothesized that during goal-directed hemodynamic therapy (GDHT), propofol anesthesia would be associated with a higher total HBF as compared with sevoflurane anesthesia. To address this question, we compared the effects of a propofol-based anesthesia versus a sevoflurane-based anesthesia on HBF and pressure in the portal and caval vein in patients undergoing pancreaticoduodenectomy. We chose this type of surgery because – in contrast to hepatic surgical procedures – pancreaticoduodenectomy is a standardized procedure without potential anticipated severe hemodynamic disturbances. In addition, during the surgical procedure there is an easy access to the hepatic blood vessels.

## Methods

### Design and patients

**Fig. 1:**
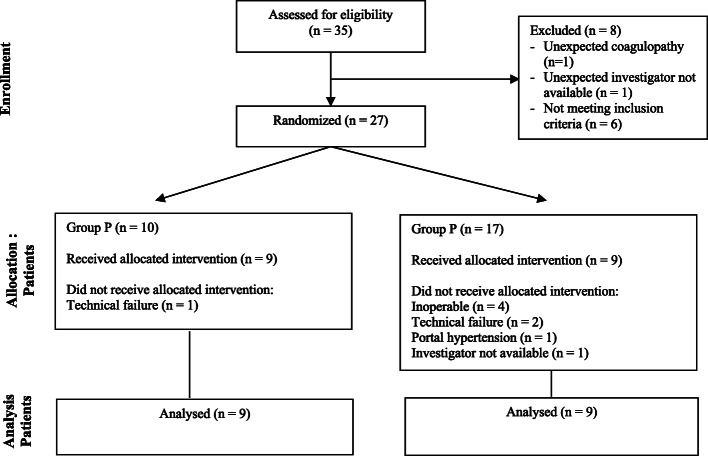
CONSORT. CONSORT flow diagram

The study was approved by the ethical committee of the University Hospital Ghent (AGO/2017/002 – EC/2017/0164) and registered under EudraCT number: 2017–000071-90. This study adheres to the CONSORT guidelines, an additional file with the CONSORT diagram is available (Fig. 1). Adult patients (age > 18 years) of both gender scheduled for a pancreaticoduodenectomy (Whipple’s procedure) in Ghent University Hospital and with an American Society of Anesthesiologists (ASA) physical status of I to III were included. Exclusion criteria were allergy to the medication, renal insufficiency (serum creatinine > 2 mg dL^− 1^), severe heart failure (ejection fraction < 25%), pre-operative hemodynamic instability, atrial fibrillation, sepsis, body mass index > 40 kg m^− 2^, severe coagulopathy (INR > 2), thrombocytopenia (< 80 × 10^3^ μL^− 1^) or history of severe postoperative nausea and vomiting (PONV).

After written informed consent, patients were randomly allocated to two groups. Group P received total intravenous anesthesia using a propofol target controlled infusion (TCI), group S received inhalation anesthesia using sevoflurane. An anesthesia co-worker, not involved in the study, performed a simple randomization using sealed pre-numbered envelopes. After randomization of all patients, drop-outs which occurred during the trial were replaced in order of their appearance.

Primary objective was to compare the effect of propofol (group P) and sevoflurane (group S) on arterial, portal and total HBF and on the caval and portal vein pressure during pancreaticoduodenectomy. The secondary objectives were to compare the need for inotropic and vasopressor support, the amount of fluids administered, plasma lactate levels and blood loss during surgery between both groups.

### Anesthetic procedure

All patients received standard anesthesia care according to the departmental protocol. Patients received ASA standard anesthesia monitoring. The departmental protocol for this type of procedures includes placement of an epidural catheter for postoperative analgesia. This catheter was placed before induction of anesthesia but only used after all experimental measurements had been performed, which was at the end of surgery. Depth of anesthesia was measured using Bispectral Index (BIS™, Covidien, MA, USA) monitoring and titrated to remain between 40 and 60. After induction of anesthesia, central venous and arterial catheters were placed. A 5-Fr PiCCO catheter (Maquet, Getinge Group, Germany) placed in the left femoral artery was used for the additional hemodynamic assessment in the current study.

Before induction of anesthesia 4 mg intravenous dexamethasone was administered for prevention of PONV. Induction and maintenance of general anesthesia differed in both groups. In group S, induction of anesthesia was obtained with propofol 1–2 mg kg^− 1^ until loss of consciousness. Anesthesia was maintained with sevoflurane. In group P, induction and maintenance were performed using propofol TCI (Schnider Model), starting at an effect site concentration of 5.0 mcg ml^− 1^. In both groups, anesthesia was titrated to obtain a BIS between 40 and 60. For intraoperative analgesia, TCI remifentanil (Minto Model) was used in both groups. TCI remifentanil was started at an effect site concentration of 5 ng ml^− 1^ and titrated according heart rate and blood pressure. Neuromuscular blockade was achieved using rocuronium, 1 mg kg^− 1^ at induction and intermittent boluses during surgery. Before each experimental measurement, an additional bolus of rocuronium 10 mg was given. After tracheal intubation and lung recruitment, mechanical ventilation was started with a tidal volume 6–8 ml kg^− 1^ ideal body weight, respiratory rate 12–14 min^− 1^ and a positive end-expiratory pressure of 5 cmH_2_O. Ventilation was adjusted according to the data of the arterial blood gas analysis. All patients received an individualized goal-directed hemodynamic therapy (GDHT) according to the departmental written procedure. A baseline crystalloid infusion (Plasmalyte A, Baxter S.A., Lessines, Belgium) of 3 ml kg^− 1^ h^− 1^ was administered. The hemodynamic goal was a cardiac index (CI) > 2.2 L min^− 1^ m^− 2^ with a mean arterial pressure (MAP) > 60 mmHg and a pulse pressure variation (PPV) < 12%. When PPV was > 12% a bolus of 200 ml colloid (Volulyte A, Fresinius Kabi NV, Schelle Belgium) was administered. When CI was > 2.2 L min^− 1^ m^− 2^ in the presence of a MAP < 60 mmHg, a noradrenaline infusion was started at 0.1 mcg kg^− 1^ min^− 1^ and titrated according to the MAP. To temporarily bridge the latency of effect the noradrenaline infusion, boluses of ephedrine 3 mg were administered when heart rate was less than 60 beats per minute or phenylephrine 0.1 mg, if heart rate was > 60 beats/min. At the end of surgery, all patients received 1 g paracetamol and 10–15 ml ropivacaine 0.15% epidurally for postoperative analgesia. A nerve stimulator was used to assess the evoked muscle response with double-burst-stimulation (DBS) or train-of-four (TOF). Reversal of neuromuscular block was done with sugammadex, guided by the twitch response to DBS or TOF.

### Measurements

Hemodynamic variables were measured using Pulsioflex™ (Maquet, Getinge Group, Germany). After placement of the 5-Fr arterial catheter in the femoral artery, the pulse contour analysis was calibrated using 3 boluses of 20 ml of cold saline. The hemodynamic variables measured were heart rate (HR), central venous pressure (CVP), MAP, CI and PPV. To assess the performance of the GDHT protocol, we calculated the percentage of time, during which the hemodynamic goals were within the limits of the targets set (CI > 2.2 L min^− 1^ m^− 2^ with PPV < 12% and MAP > 60 mmHg).

During surgery, 3 flow measurements were performed by the surgeon, at predefined times, while systemic hemodynamic variables were recorded. Pancreaticoduodenectomy is a standardized surgical procedure, which we divided in three different stages. The first flow measurements were made after transection of the gastro-duodenal artery (T1). The second flow measurement (T2) was performed after pancreatectomy. The last flow measurement (T3) was performed before surgical reconstruction and minimal 10 min after T2. Blood flow measurements at the hepatic artery and portal vein were obtained using perivascular ultrasound transit time flow probes (TTFM, Medi-Stim AS, Oslo, Norway) [19]. Different probe sizes were used according to the type and size of the vessel (range 2–12 mm). Blood flow was expressed in ml min^− 1^. At the same time, the pulsatility index (PI) was calculated by the TTFM. PI quantifies pulsatility of a blood flow wave which represents vascular resistance of the blood vessel downstream. PI is calculated by maximum volumetric peak flow minus minimum volumetric peak flow divided by mean volumetric volume [20]. Simultaneously with flow measurements, additional pressure measurements were performed in the portal and caval vein. A 25-gauge needle was directly placed in the vein and connected to a pressure transducer. Systemic hemodynamic, regional hepatic flow and portocaval pressure measurements were performed simultaneously during apnea to minimize the effect of ventilation. The relative blood flow over the hepatic artery or portal vein was calculated by dividing arterial or portal HBF by CO.

### Statistical analysis

To the best of our knowledge, no previous studies are available comparing the effect of propofol and sevoflurane on HBF. Therefore, we could not rely on previous publications to determine the exact sample size needed to compare the effects of both anesthetics on HBF. As such, the current study is also a feasibility study and the information provided can be used for sample size calculation of future studies assessing HBF using TTFM. The publication of Sand Bown et al. [21] was used to define a clinically relevant reduction of HBF. Based on this publication, a 30% reduction in arterial and portal HBF was considered clinically significant. G*Power 3.1.9.2 was used to calculate the sample size [22]. For an alpha error of 5%, a beta error of 20%, SD of 0.25 and an effect size F of 0.6, each group necessitated 9 patients to detect a flow reduction of 30%. After testing for normal distribution with the Shapiro-Wilk normality test, data between both groups were compared using a two-way ANOVA for repeated measurements, or its non-parametric equivalent where appropriate. Pairwise comparisons were done using paired t-test with Bonferroni correction for significance.

Numbers of patients necessitating vasopressor support were compared using Fisher exact test. All statistical tests were performed using R (version 3.3.3) [23].

## Results

### Patient characteristics

Between June 2017 and January 2018, a total of 35 patients were assessed for eligibility to participate in the study. Six patients were excluded based on the exclusion criteria. Twenty- nine patients were included. Two patients were additionally excluded due to unexpected coagulopathy and investigator unavailability. A total of 27 patients were randomized of whom 10 in group P and 17 in group S. In group S, 8 patients dropped out because of in-operability (*n* = 4), technical failure of the registration device (*n* = 2), unexpected portal hypertension (*n* = 1) and investigator unavailability (*n* = 1). In group P, 1 patient dropped out due to technical failure of the registration device. Finally, data of 9 patients in each group were analyzed (Fig. 1). Patient characteristics are listed in Table 1. Both groups were comparable with respect to age, gender, length, weight, BMI, ASA physical status, pre-operative blood pressure and heart rate, and smoking status.

**Table 1:** Patient characteristics

	Propofol Group (*n* = 9)	Sevoflurane Group (*n* = 9)
Age (year)	63.6 (5.4)	63.9 (12.0)
Gender (F/M)	4/5	3/6
Height (cm)	169.1 (8.8)	169.8 (7.9)
Weight (kg)	72.0 (7.5)	67.4 (9.9)
BMI (kg m^−2^)	25.2 (2.5)	23.3 (2.5)
Smoker	1	5
ASA class (I/II/III)	1/3/5	0/6/3

Data are presented as mean (SD). F/M Female/male ratio, Body mass index (BMI), American Society of Anesthesiologist physical status (ASA)

### Hemodynamic variables

**Fig. 2:**
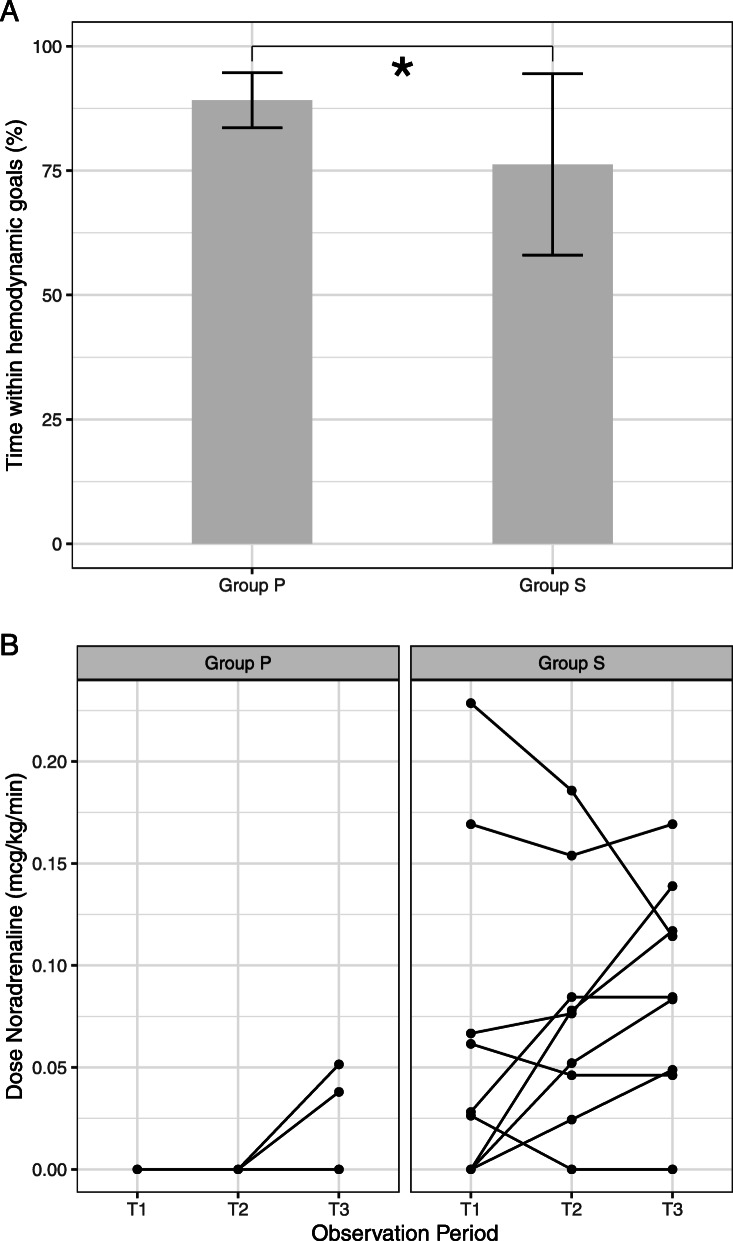
**a**: Maintenance of hemodynamic targets. Efficacy of goal-directed hemodynamic therapy during procedure: percentage of time within hemodynamic goals as defined in the departmental protocol between propofol titrated-patients (group P) and sevoflurane-titrated patients (group S). * *P* < 0.05. **b**: Noradrenaline infusion. Noradrenaline infusion related to observation periods

Hemodynamic variables are listed in Table 2. All patients received individualized GDHT as described above. The pre-set hemodynamic targets were obtained in both groups, but MAP was lower in group S (*p* = 0.01). Successful achievement of the hemodynamic targets, as defined by the cumulative time within pre-set hemodynamic goals were met, and expressed as a percentage of total study duration, it was higher in group P (*p* = 0.046) (Fig. 2a). In group P mean percentage of time in range was 89% (SD 5.5%) while in group S, a mean of 76% (SD 18.2%) was achieved. The total dose of vasopressors needed to obtain these pre-set targets however was higher in group S than for group P ephedrine respectively 10.4 mg (SD 5.6 mg) versus 5.3 mg (SD 3.3 mg) (*p* = 0.04) and noradrenaline infusion 2809 mcg (SD 2197 mcg) versus 227 mcg (SD 237 mcg) (*p* 0.0004) (Fig. 2b). All patients required noradrenaline in group S, as compared to only 3 patients in group P (*p* = 0.009). A rise in blood lactate levels over time was observed in both groups (*p* = 0.0003) but the increase was significantly more pronounced in group S (*p* = 0.04).

**Table 2:** Hemodynamic data

		Propofol Group (*n* = 9)	Sevoflurane Group (*n* = 9)	Between group difference
MAP (mmHg)	T1	82 (9)	69 (10)	13 [4–22]^a^
	T2	76 (5)	74 (9)	1 [−7–9]
	T3	82 (5)	76 (9)	6 [−2–13]
HR (bpm)	T1	75 (12)	78 (14)	-2 [− 19–14]
	T2	79 (10)	80 (11)	-1 [− 15–13]
	T3	77 (8)	79 (12)	-2 [−16–11]
CVP (mmHg)	T1	5 (2)	5 (2)	0 [−2–2]
	T2	5 (1)	5 (4)	-1 [−4–2]
	T3	4 (2)	6 (2)	-2 [−4–1]
CI (L.min^−1^.m^−2^)	T1	2.7 (0.4)	3.1 (0.8)	−0.3 [−1.1–0.4]
	T2	3.0 (0.5)	3.3 (0.6)	−0.3 [−1–0.4]
	T3	3.0 (0.4)	3.2 (0.6)	−0.2 [− 0.9–0.4]
SVR (dyn.sec.cm^− 5^)	T1	1267 (231)	991 (230)	276 [−52–604]
	T2	1084 (209)	1000 (316)	84 [−301–469]
	T3	1168 (178)	1031 (261)	136 [−166–439]
PPV (%)	T1	9 (4.0)	8 (4.6)	1.2 [−3.6–6.1]
	T2	9 (1.7)	10 (5.6)	−0.9 [−4.9–3.1]
	T3	8 (3.0)	8 (5.8)	0.1 [−3.9–4.1]
Lactacte (mg.dL^−1^)	T1	8.9 (1.5)	12.2 (3.8)	−3.3 [− 6.0 – − 0.6]^a^
	T2	10.1 (2.7)^b^	16.3 (6.8)^b^	−6.2 [− 10.9 – − 1.6]^a^
	T3	10.5 (2.9)^b^	18.2 (8.4)^b^	−7.7 [− 13.9 – − 1.5]^a^
P_a_CO_2_ (mmHg)	T1	42 (4.5)	42 (5.9)	0.1 [−5.2–5.4]
	T2	41 (6.1)	43 (4.8)	−2.1 [−8.2–4.1]
	T3	42 (3.3)	42 (5.1)	−0.3 [−3.8–4.5]
pH	T1	7.36 (0.05)	7.34 (0.05)	0.02 [−0.04–0.08]
	T2	7.35 (0.05)	7.33 (0.05)	0.01 [−0.04–0.07]
	T3	7.34 (0.05)	7.35 (0.06)	−0.01 [− 0.06–0.03]

Data are presented as mean (SD). *MAP* Mean arterial pressure, *HR* Heart rate, *CVP* Central venous pressure, *CI* Cardiac index, *SVR* Systemic vascular resistance, *PPV* Pulse pressure variation, *P*_*a*_*CO*_*2*_ Arterial carbon dioxide tension. Bonferroni corrected significance are marked as ^a^ for between group comparisons and ^b^ for significant within group difference compared to T1

### Fluid management

Intraoperative characteristics are listed in Table 3. The total amount of administered crystalloids was similar between group P and group S respectively 1974 ml (SD 440 ml) versus 2308 ml (SD 471 ml) (*p* = 0.14). The total amount of administered colloids was similar between both groups, 1067 ml (SD 500 ml) for group P versus 1078 ml (441 ml) for group S (*p* = 0.96). Surgical time was significantly longer in group S, 534 min (SD 98 min) compared with group P, 465 min (SD 68 min) (*p* = 0.0002). This was related to variability in the time to obtain a diagnosis from the intraoperative frozen section. However, there was no difference in the delivered amount of crystalloid per minute between both groups, 4.7 ml.min^− 1^ (SD 2.3 ml.min^− 1^) for group S compared with 4.3 ml.min^− 1^ (SD 0.8 ml.min^− 1^) for group P (*p* = 0.63). Urinary output was similar in both groups, respectively 779 ml (SD 602 ml) for group S compared with 463 ml (SD 198 ml) (*p* = 0.17). Also, blood loss was comparable in both groups (*p* = 0.47).

**Table 3:** Intraoperative characteristics

	Propofol Group (*n* = 9)	Sevoflurane Group (*n* = 9)
Crystalloids (ml)	1974 (440)	2308 (471)
Crystalloids (ml.min^− 1^)	4.3 (0.8)	4.7 (2.3)
Colloids (ml)	1067 (500)	1078 (441)
Blood loss (ml)	567 (212)	689 (448)
Urinary Output (ml)	463 (198)	779 (602)
Ephedrine (mg) *	5.3 (3.3)	10.3 (5.6)
Phenylephrine (mcg)	0.16 (0.11)	0.37 (0.38)
Noradrenaline (mcg) *	227 (237)	2809 (2197)
Patients requiring vasopressor support	3	9
Surgical time (min) *	465 (68)	534 (98)
Remifentanil (mcg)	3915 (1520)	3826 (1068)
Patients receiving Somatostatin infusion	4	5

Intraoperative characteristics are expressed in mean (SD)

* = statistically significant difference (*p* < 0.05) between group comparison

### Flow measurements

Flow measurements are summarized in Table 4. Total HBF was similar in both groups at all points of measurement (*p* = 0.76). There was no difference in portal HBF (*p* = 0.85) and arterial HBF (*p* = 0.70) between both groups at all time points. There was no difference between the relative blood flow in the hepatic artery (*p* = 0.67) and in the portal vein (*p* = 0.85) between both groups. The ratio portal over arterial HBF also showed no difference between groups (*p* = 0.22). Portal and caval vein pressures were similar in both groups at all measurement times. The PI of both portal vein (*p* = 0.38) and hepatic artery (*p* = 0.61) showed no difference between groups.

**Table 4:** Hepatic blood flow and pressures

		Propofol Group (*n* = 9)	Sevoflurane Group (*n* = 9)	Between group difference
Total HBF (ml.min^−1^)	T1	997 (344)	1003 (411)	−5 [− 395–384]
	T2	937 (231)	860 (318)	77 [− 272–426]
	T3	998 (264)	943 (225)	55 [− 252–362]
Portal HBF (ml.min^− 1^)	T1	760 (275)	790 (317)	−30 [− 352–291]
	T2	687 (203)	612 (218)	75 [− 163–313]
	T3	714 (210)	704 (137)	10 [− 234–254]
Arterial HBF (ml.min^− 1^)	T1	237 (150)	212 (138)	25 [− 130–180]
	T2	249 (110)	247 (199)	2 [−212–216]
	T3	284 (101)	239 (149)	45 [−110–200]
Relative Total HBF (% of CO)	T1	20.0 (6.0)	19.9 (12.1)	0.1 [−7.4–7.6]
	T2	17.4 (3.4)	15.5 (7.6)	1.8 [−5.2–8.9]
	T3	18.4 (4.1)	17.4 (6.7)	1.0 [−4.8–6.7]
Relative Portal HBF (% of CO)	T1	15.2 (5.2)	15.9 (9.4)	−0.6 [−8.1–6.9]
	T2	12.8 (3.7)	11.1 (5.3)	1.6 [−3.7–7.0]
	T3	13.1 (3.8)	12.8 (3.8)	0.3 [−3.7–4.3]
Relative Arterial HBF (% of CO)	T1	4.8 (2.8)	4.1 (3.4)	0.7 [−1.4–2.8]
	T2	4.6 (1.9)	4.4 (3.7)	0.2 [−3.6–4.0]
	T3	5.3 (2.0)	4.6 (3.5)	0.7 [−2.6–4.0]
Portal Vein Pressure (mmHg)	T1	10 (6)	9 (3)	1 [−4–7]
	T2	6 (3)	10 (4)	−4 [−7–0]
	T3	8 (4)	9 (5)	0 [−4–3]
Caval Vein Pressure (mmHg)	T1	6 (3)	5 (3)	1 [−2–4]
	T2	5 (3)	7 (4)	−1 [−5–2]
	T3	7 (4)	7 (3)	0 [−5–4]
PI Portal Vein	T1	0.4 (0.2)	0.5 (0.3)	−0.1 [−0.4–0.2]
	T2	0.3 (0.2)	0.5 (0.3)	−0.2 [− 0.4–0]
	T3	0.5 (0.2)	0.5 (0.2)	0 [−0.1–0.2]
PI Hepatic Artery	T1	1.4 (0.9)	1.7 (0.9)	−0.3 [− 0.9–0.4]
	T2	1.4 (0.6)	1.5 (0.7)	−0.1 [− 0.8–0.5]
	T3	1.4 (0.7)	1.5 (0.5)	− 0.1[− 0.7–0.6]

Data are presented as mean (SD) for both groups. Mean estimated between group differences with their confidence intervals (95%) are provided in the right column. No Bonferroni corrected significant difference (*p* < 0.05) were found for between or within group comparisons. Hepatic blood flow (HBF). PI = pulsatility index. CO = cardiac output. No significant differences (*p* < 0.05) were found for between or within group comparisons

## Discussion

In this study we compared the effect of a propofol- and sevoflurane-based anesthesia on HBF during GDHT. Our results showed that portal, arterial and total HBF were similar in propofol- and sevoflurane-anesthetized patients. Due to the application of a GDHT protocol similar hemodynamic variables were observed in both groups. However, patients in group S required a significantly higher administration of vasopressor to maintain adequate MAP.

To our knowledge there are no previous human trials, assessing and comparing HBF with direct flow measurements under propofol- and sevoflurane-based anesthesia.

Clinical practice guidelines on liver transplantation are lacking advice for the choice of anesthetic technique for maintenance [24]. Previous studies have suggested that sevoflurane compared to propofol may attenuate the effects of ischemia-reperfusion injury after liver resection [25]. However, a similar study comparing effects of propofol on sevoflurane on hepatic graft survival yielded no different effects between both anesthetic agents [26]. Maintaining adequate HBF is important for allograft [2–4] and patient survival [5, 6]. Yet, potential effects of anesthetic agents on HBF in the clinical setting remain largely unexplored. Both sevoflurane and propofol have an effect on HBF [7]. Conflicting results about the effect of propofol on HBF have been described. Previous studies have suggested that propofol increases total HBF. However, the putative mechanism for this increase in HBF differs between the studies. A study in rats showed an increase in total HBF by an increase of both arterial and portal HBF. Propofol reduced hepatic arterial resistance and portal venous resistance in an identical manner [14]. A study in dogs showed similar results. However, in this study, there was only a transient increase in total HBF by propofol which was mediated primarily by an increased arterial HBF [13]. A study in rabbits showed an increased total HBF with propofol, primarily by an increased portal HBF [15]. Conversely, one study in sheep showed a reduction in total HBF [27]. Only one human study was performed. In this study, desflurane and propofol were compared in 20 patients using a cross-over design. Total HBF was significantly higher in propofol-treated patients compared to desflurane-treated patients [16]. The mechanism behind the observed effects of propofol on HBF remains unclear. It was assumed that the metabolization of propofol increases hepatic oxygen consumption. To maintain hepatic oxygen balance, there would be then a compensatory increased oxygen delivery primarily by increasing portal HBF [14, 15].

The effect of sevoflurane on HBF remains unclear. Animal studies suggested that sevoflurane has only minimal effects on total HBF. A study in dogs showed that sevoflurane resulted in a hepatic vasodilation with a reduction in portal HBF at 1.2 and 2.0 MAC but a significant increased arterial HBF was only seen at 2.0 MAC [17]. Other animal studies confirmed this finding. Sevoflurane maintained total HBF, and although portal HBF was reduced, arterial HBF increased, resulted in sufficient HBF to maintain hepatic oxygen delivery [18, 28]. Results from human studies are conflicting. Hongo et al. showed a reduction in total HBF in sevoflurane but Kanaya et al. on the contrary found no effect on HBF with sevoflurane [29, 30].

The previous studies, both animal and human, used different techniques to measure arterial, portal and total HBF. HBF can be measured both directly and indirectly [31]. Indirect measurements are less invasive but also less accurate. Examples of indirect measurements are radio-labelled microspheres [14] or indicator substance such as sodium bromsulphthalein [27] and the indocyanine green (ICG) clearance test [16, 29, 30]. Propofol interacts with ICG and inhibits the hepatic clearance of ICG which may consequently lead to an underestimation of true HBF [32, 33]. Recently, total HBF was measured indirectly by calculating blood flow at the hepatic vein using transesophageal echocardiography [34]. Direct measurement of HBF is a fast and accurate technique but is also more invasive. Previous studies used Doppler or electromagnetic flow probes which were directly placed around the hepatic artery and portal vein [13, 15, 17].

During liver transplantation, assessment of the graft blood flow by TTFM plays an important role in the assessment of the survival chances of the allograft [35, 36]. If flow measurements are needed, TTFM is very reliable and is considered to be the ¨gold standard¨ for measuring blood flow [19]. As our study demonstrated, measuring HBF using TTFM is feasible in a clinical steady state.

The results of the present study should be interpreted within the constraints of the methodological protocol. First, as a predefined GDHT was used to maintain patient’s hemodynamic stability, the current data should not be interpreted as a direct independent effect of both propofol and sevoflurane on the hepatic circulation. Indeed, hemodynamic targets were achieved in both groups, but to achieve this, a significantly higher vasopressor support was needed in sevoflurane-titrated patients, while propofol-titrated patients had higher MAP, well above target MAP without vasopressor support. As both groups were comparable of depth of anesthesia, a possible explanation could be a more profound vasodilation with sevoflurane than with propofol. As this vasodilating effect was compensated by the vasopressor therapy, it cannot be excluded that at the same time a vasodilatory effect at the level of the hepatic circulation was also blunted. In the present study noradrenaline was used to maintain adequate MAP. The effect of noradrenaline on HBF during surgery remains complex and unclear. The splanchnic circulation has a wide variety and distribution of adrenergic receptors [37] and therefore noradrenaline may affect HBF. Previous animal studies suggested that noradrenaline reduced HBF [38], primarily by reducing arterial HBF [39]. However, a recent study in pigs showed that noradrenaline infusion - used to correct hypotension - did not affect HBF during abdominal surgery [40]. The current observations do not allow to make inferences of potential independent effects of noradrenaline on HBF. Interestingly, lactate levels in the present study were higher in group S. Although we do not have a straightforward explanation for this phenomenon, it might be seen as indication that despite the GDHT-related stability in hemodynamic variables, global tissue oxygenation was jeopardized more than in group P.

Secondly, the data obtained may have been influenced by other factors related to intra-operative patient care. A total of 9 patients received – on surgical indication - somatostatin at 250 mcg h^− 1^ (4 in group P and 5 in group S) to reduce pancreatic secretion. Previous animal studies suggested that somatostatin may affect portal HBF and portal pressure primarily in the presence of portal hypertension [41, 42]. We cannot exclude that the use of somatostatin had an influence on the results, but the number of patients treated were equally divided between both groups. In addition, a post-hoc sub-analysis comparing patients with and without somatostatin treatment revealed no differences in hemodynamic or hepatic flow profiles. Thirdly, selecting the correct size of the probe is of crucial importance to obtain reliable flow data, as the use of an oversized probe may lead to overestimation of the blood flow [43]. In our institution TTFM is a routinely used procedure during major liver surgery and liver transplantation. The size of the probe was meticulously assessed by the participating surgeons who are highly experienced in the use of this technique. Fourthly, a total of 9 patients dropped out during the trial. These patients were replaced after randomization in order of their dropout appearance. This may impose a risk for allocation bias. As most dropouts occurred due to inoperability, this could not be influenced by the researcher. Replacement of dropouts was done in order of their dropout appearance, which could not be influenced by the researcher. Therefore, the risk for allocation bias as such seems limited. Fifthly, no previous studies were available to assess differences in HBF between sevoflurane- and propofol-anesthetized patients. Therefore, we could not rely on previous publications to determine the exact sample size needed to compare the effects of both anesthetics on HBF and we relied on the publication of Sand Bown et al. [21] to determine the clinically relevant reduction of HBF. However, a reduction of 30% in portal and arterial HBF is probably an overestimation of the real effect size. This may impose a risk for insufficient power of the study. To address this issue, we conducted a post-hoc power analysis with our current results. We observed a mean total HBF for propofol of 977 ml.min^− 1^ (SD 260 ml.min^− 1^) and for sevoflurane of 935 ml.min^− 1^ (SD 300 ml.min^− 1^). When using the results of Meierhenrich [16], who had an effect size f of 0.54, we calculated a post hoc power of 75% which is slightly lower than the a priori set power of 80%. As such, the current study should be considered as a pilot study, performed to check the feasibility of assessing HBF during goal-directed hemodynamic therapy and to provide clinically relevant data on HBF under anesthesia, which may be used, to explore effect size assessments in future trials.

## Conclusion

The results of the present study indicate that when applying a GDHT, aiming at stable hemodynamic variables, HBF during propofol- and sevoflurane-based anesthesia was similar. However, to maintain these identical hemodynamic goals, sevoflurane-anaesthetized patients necessitated a significantly higher need for vasopressor support and blood lactate levels were higher in comparison to patients receiving propofol-based anesthesia.

## Data Availability

The datasets used and/or analyzed during the current study are available from the corresponding author on reasonable request.

## Abbreviations

BMI: Body Mass Index
CI: Cardiac Index
CO: Cardiac Output
CVP: Central Venous Pressure
DBS: Double Burst Stimulation
GDHT: Goal-directed Hemodynamic Therapy
HBF: Hepatic Blood Flow
HR: Heart Rate
MAP: Mean Arterial Pressure
PI: Pulsatility index
PONV: Postoperative Nausea and Vomiting
PPV: Pulse Pressure Variation
PVP: Portal Venous Pressure
PVR: Portal Venous Resistance
SVR: Systemic Vascular Resistance
TCI: Target Control Infusion
TOF: Train-of-four
TTFM: Transit Time Flow Measurement
